# Supplementary material for: The Anti-Acne Potential and Chemical Composition of Knautia drymeia Heuff. and Knautia macedonica Griseb Extracts
Source: Int J Mol Sci. 2023 May 24;24(11):9188. doi: 10.3390/ijms24119188 (PMC10252809; doi:10.3390/ijms24119188)
Supplement: Supplementary file 1 [file ijms-24-09188-s001.zip › ijms-2388752-supplementary.pdf]

Table S1. High-resolution mass spectrometry (HR-MS) of $[M-H]^-$ ion and MS^2 data.

Peak No.	Name of Compound	$[M-H]^-$	MS^2	Theoretical Mass $[M-H]^-$ (Da)	Experimental Mass $[M-H]^-$ (Da)	Δ mDa	Δ ppm	Elemental Composition
1	Gallic acid	169	69, 79, 81, 97, 124, 125b	169.01370	169.01367	-0.03	0.18	$C_7H_6O_5$
2	Neochlorogenic acid	353	135, 179, 191b	353.08726	353.08729	0.03	0.08	$C_{16}H_{18}O_9$
3	Chlorogenic acid	353	179, 191b	353.08726	353.08731	0.05	0.14	$C_{16}H_{18}O_9$
4	*p*-Hydroxybenzoic acid	137	92, 93, 136, 137b	137.02387	137.02391	0.04	0.29	$C_7H_6O_3$
5	Cryptochlorogenic acid	353	135, 173b, 179, 191	353.08726	353.08718	-0.08	0.23	$C_{16}H_{18}O_9$
6	Caffeic acid	179	97, 107, 135b	179.03444	179.03440	-0.04	0.22	$C_9H_8O_4$
7	Syringic acid	197	85, 121b, 138, 153, 181, 197	197.04500	197.04507	0.07	0.36	$C_9H_{10}O_5$
8	*p*-Coumaric acid	163	119, 162b	163.03952	163.03944	-0.08	0.49	$C_9H_8O_3$
9	*o*-Coumaric acid	163	119, 162b	163.03952	163.03957	0.05	0.31	$C_9H_8O_3$
10	Ferulic acid	193	73, 89b, 106, 117, 133, 134, 179	193.05009	193.05012	0.03	0.16	$C_{10}H_{10}O_4$
11	3,5-Di-*O*-caffeoylquinic acid	515	191, 335, 353b, 515	515.11896	515.11891	-0.05	0.10	$C_{25}H_{24}O_{12}$
12	Vanillic acid	167	108 ,123 ,152,167b	178.04774	178.04769	-0.05	0.28	$C_8H_8O_4$
13	*m*-Hydroxybenzoic acid	137	92, 93, 136, 137b	137.02387	137.02383	-0.04	0.29	$C_7H_6O_3$
14	Salicylic acid	137	92, 93, 136, 137b	137.02387	137.02381	-0.06	0.44	$C_7H_6O_3$
15	Kaempferol-3-*O*-rutinoside	593	85, 287b, 447	593.15065	593.15075	0.10	0.17	$C_{27}H_{30}O_{15}$
16	Rutin	609	151, 179, 301b, 463, 609	609.18195	609.18192	-0.03	0.05	$C_{27}H_{30}O_{16}$
17	Luteolin-7-*O*-rutinoside	593	85, 287b, 447	593.15065	593.15069	0.04	0.07	$C_{27}H_{30}O_{15}$
18	Quercetin-3-*O*-*β*-D-(2″-*O*-*β*-D-xylosyl) galactoside	595	151, 179, 301, 433b, 595	595.12992	595.12999	0.07	0.12	$C_{26}H_{28}O_{16}$
19	Naringin	579	145, 151, 271, 579b	579.17139	579.17128	-0.11	0.19	$C_{27}H_{32}O_{14}$
20	Rhoifolin	577	269b, 577	577.15574	577.15577	0.03	0.05	$C_{27}H_{30}O_{14}$
21	Isorhoifolin	577	269b, 577	577.15574	577.15570	-0.04	0.07	$C_{27}H_{30}O_{14}$
22	Hyperoside	463	151, 179, 255, 271, 301b	463.08766	463.08769	0.03	0.06	$C_{21}H_{20}O_{12}$

23	Isoquercitrin	463	151, 179, 192, 301, 461, 463b	463.08766	463.08761	-0.05	0.11	$C_{21}H_{20}O_{12}$
24	Quercitrin	447	151, 243, 255, 271, 300b, 301, 447	447.09274	447.09279	0.05	0.11	$C_{21}H_{20}O_{11}$
25	Quercetin-3-*O*-glucuronide	477	151, 301b , 477	477.06692	477.06687	-0.05	0.10	$C_{21}H_{18}O_{13}$
26	Orientin	447	285, 297, 327b, 357, 447	447.09274	447.09283	0.09	0.20	$C_{21}H_{20}O_{11}$
27	Homoorientin	447	285, 297, 327b, 357, 447	447.09274	447.09261	-0.13	0.29	$C_{21}H_{20}O_{11}$
28	Luteolin-7-*O*-glucuronide	461	216b, 285, 461	461.07201	461.07208	0.07	0.15	$C_{21}H_{18}O_{12}$
29	Kaempferol-3-*O*-glucuronide	461	113, 229, 285b, 461	461.07201	461.07215	0.14	0.30	$C_{21}H_{18}O_{12}$
30	Apigenin-7-*O*-glucuronide	445	117, 149, 151, 225, 269b	445.07709	445.07703	-0.06	0.13	$C_{21}H_{18}O_{11}$
31	Luteolin-7-*O*-glucoside	447	133, 151, 175, 199, 287b, 447	447.09274	447.09263	-0.11	0.25	$C_{21}H_{20}O_{11}$
32	Kaempferol-7-*O*-glucoside	447	257, 285b, 447	447.09274	447.09278	0.04	0.09	$C_{21}H_{20}O_{11}$
33	Kaempferol-3-*O*-glucoside	447	257, 285b, 447	447.09274	447.09287	0.13	0.29	$C_{21}H_{20}O_{11}$
34	Isovitexin	431	283, 311b, 323, 341	431.09783	431.09780	-0.03	0.07	$C_{21}H_{20}O_{10}$
35	Vitexin	431	283, 311b, 323, 341	431.09783	431.09794	0.11	0.26	$C_{21}H_{20}O_{10}$
36	Apigetrin	431	117, 149, 151, 225, 269b, 431	431.09783	431.09797	0.14	0.32	$C_{21}H_{20}O_{10}$
37	Myricetin	317	107, 109, 137, 151, 179, 317b	317.02975	317.02974	-0.01	0.03	$C_{15}H_{10}O_{8}$
38	Rosmarinic acid	359	73, 123, 133, 135, 161b, 179, 197, 359	359.0767	359.07675	0.05	0.14	$C_{18}H_{16}O_{8}$
39	Sinapic acid	223	59, 141, 148, 164b, 178, 208, 223	223.06065	223.06069	0.04	0.18	$C_{11}H_{12}O_{5}$
40	Quercetin	301	151, 179b, 192, 209	301.03483	301.03487	0.04	0.13	$C_{15}H_{10}O_{7}$
41	Kaempferol	285	93, 97, 119b, 164, 285	285.03992	285.03983	-0.09	0.32	$C_{15}H_{10}O_{6}$
42	Luteolin	285	133, 151, 175, 199, 285b	285.03992	285.03982	-0.10	0.35	$C_{15}H_{10}O_{6}$
43	Apigenin	269	117, 149, 151, 225, 269b	269.04500	269.04502	0.02	0.07	$C_{15}H_{10}O_{5}$
44	Naringenin	271	107, 119, 151b, 177, 271	271.06065	271.06077	0.12	0.44	$C_{15}H_{12}O_{5}$
45	Galangin	269	223, 227, 269b	269.04500	269.04505	0.05	0.19	$C_{15}H_{10}O_{5}$
46	Chrysin	253	101, 127, 145, 151b, 253b	253.05009	253.05012	0.03	0.12	$C_{15}H_{10}O_{4}$
47	Pinocembrin	255	101, 129, 151b, 255	255.06574	255.06579	0.05	0.20	$C_{15}H_{12}O_{4}$

b – base peak

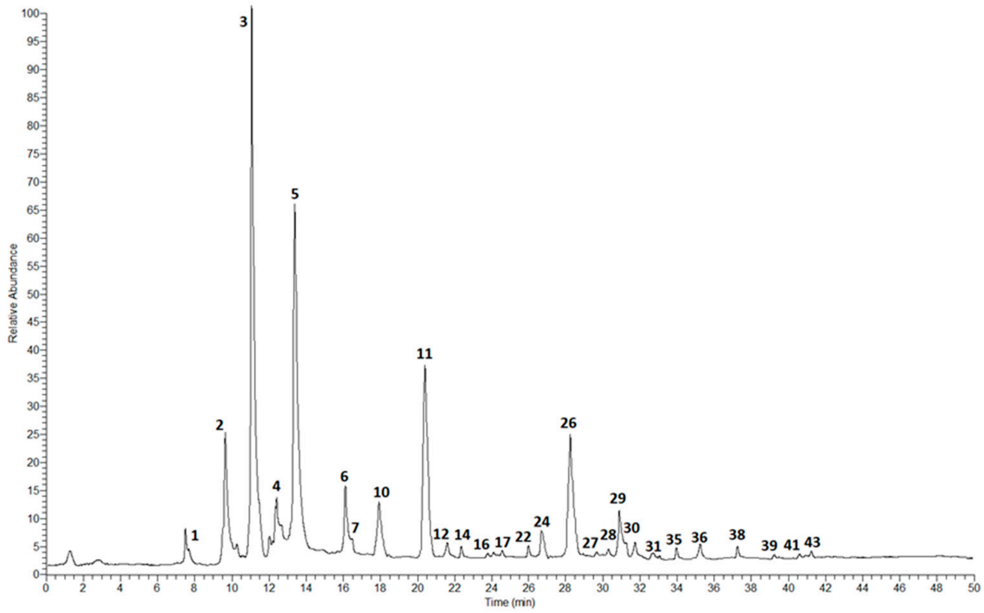

Figure S1. LC-MS chromatogram in SCAN mode for ethanol extract of *K. drymeia* (KDE).

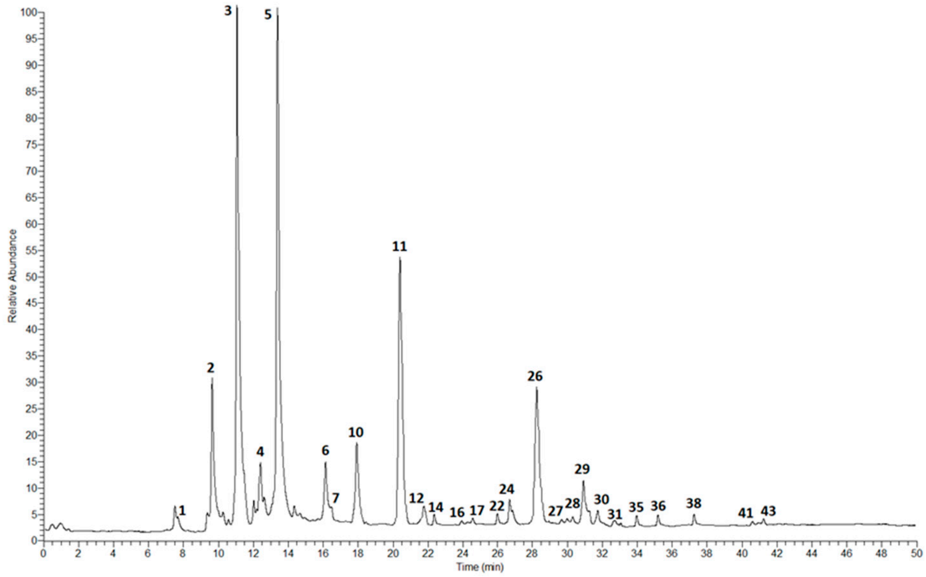

Figure S2. LC-MS chromatogram in SCAN mode for methanol-acetone-water (3:1:1) extract of *K. drymeia* (KDM).

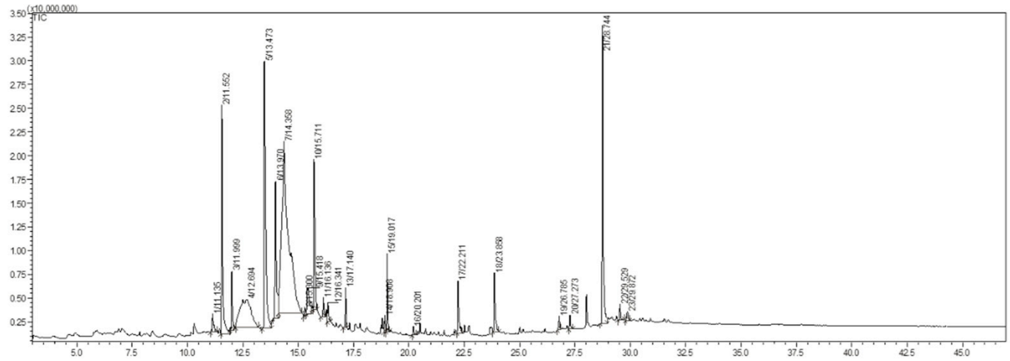

Figure S3. GC-MS chromatogram (in TIC mode) of ethanol extract of *K. drymeia* (KDE).

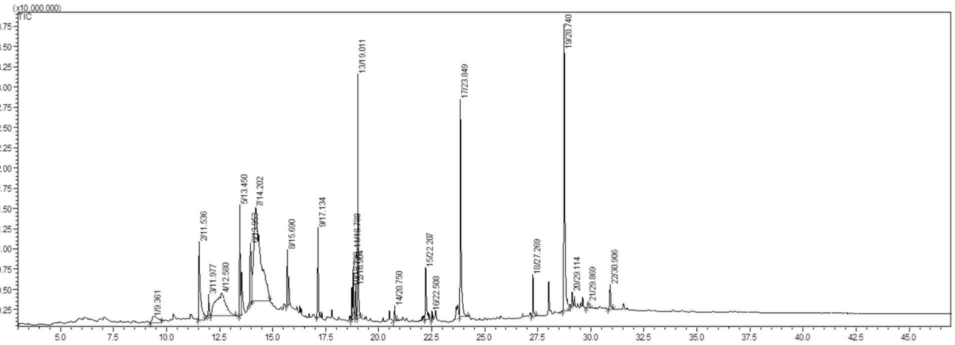

Figure S4. GC-MS chromatogram (in TIC mode) of ethanol extract of *K. macedonica* (KME).
